# Construction of a BiVO_4_/V_S_-MoS_2_ S-scheme heterojunction for efficient photocatalytic nitrogen fixation†

**DOI:** 10.1039/d3na01091k

**Published:** 2024-02-20

**Authors:** Han-Ying Luo, Zhao-Lei Liu, Meng-Ran Zhang, Yan-Fei Mu, Min Zhang

**Affiliations:** a MOE International Joint Laboratory of Materials Microstructure, Institute for New Energy Materials and Low Carbon Technologies, School of Materials Science and Engineering, School of Chemistry and Chemical Engineering, Tianjin University of Technology Tianjin 300384 China zm2016@email.tjut.edu.cn; b School of Chemistry and Chemical Engineering, Yangzhou University Yangzhou Jiangsu 225009 China 007916@yzu.edu.cn

## Abstract

Photocatalytic nitrogen (N_2_) reduction to ammonia (NH_3_), adopting H_2_O as the electron source, suffers from low efficiency owing to the sluggish kinetics of N_2_ reduction and the requirement of a substantial thermodynamic driving force. Herein, we present a straightforward approach for the construction of an S-scheme heterojunction of BiVO_4_/V_S_-MoS_2_ to successfully achieve photocatalytic N_2_ fixation, which is manufactured by coupling an N_2_-activation component (V_S_-MoS_2_ nanosheet) and water-oxidation module (BiVO_4_ nanocrystal) through electrostatic self-assembly. The V_S_-MoS_2_ nanosheet, enriched with sulfur vacancies, plays a pivotal role in facilitating N_2_ adsorption and activation. Additionally, the construction of the S-scheme heterojunction enhances the driving force for water oxidation and improves charge separation. Under simulated sunlight irradiation (100 mW cm^−2^), BiVO_4_/V_S_-MoS_2_ exhibits efficient photocatalytic N_2_ reduction activity with H_2_O as the proton source, yielding NH_3_ at a rate of 132.8 μmol g^−1^ h^−1^, nearly 7 times higher than that of pure V_S_-MoS_2_. This study serves as a noteworthy example of efficient N_2_ reduction to NH_3_ under mild conditions.

## Abstract 2

An S-scheme heterojunction (BiVO_4_/V_S_-MoS_2_) was prepared, successfully achieving N_2_ photofixation, yielding about 132.8 μmol g^−1^ h^−1^ of NH_3_ under simulated sunlight irradiation.

## Graphical abstract

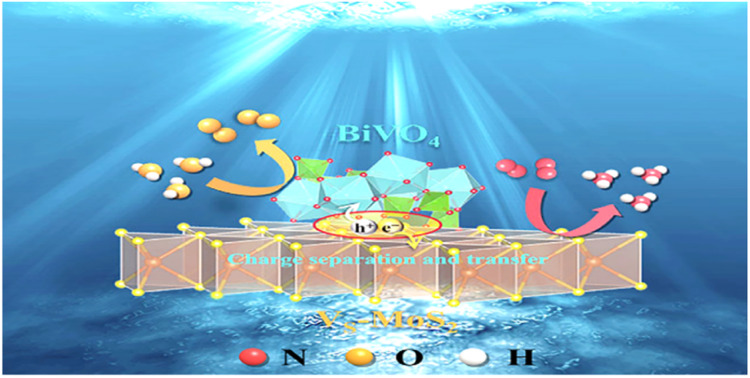

## 1. Introduction

Ammonia (NH_3_) serves as both a promising energy storage intermediary and an essential raw material affecting agricultural and industrial production.^1,2^ However, given the molecular inertness of N_2_ (dissociation energy of 945 kJ mol^−1^), the current industrial synthesis of NH_3_ predominantly relies on the Haber–Bosch process.^3,4^ This traditional method requires high temperature and pressure (300–550 °C and 15–25 MPa), resulting in significant investment costs and high energy consumption.^5,6^ Therefore, the pursuit of synthetic processes under mild conditions has become a paramount objective. Photocatalytic N_2_ fixation, employing semiconductor photocatalysts that harness sunlight as the energy source while utilizing N_2_ and H_2_O as reactants, stands out as a sustainable approach for NH_3_ production.^7–10^ Nonetheless, because of the poor N_2_ adsorption–activation and carrier separation in semiconductor photocatalysts, achieving efficient photocatalytic conversion of N_2_ to NH_3_ remains a scientific challenge.^11–13^

Efficient N_2_ adsorption–activation and good light absorption of photocatalysts are the fundamental prerequisites for realizing the efficient photoreduction of N_2_ to produce NH_3_.^14^ Consequently, the initial step involves the selection of potential semiconductor materials to reasonably design photocatalysts with suitable energy levels for N_2_ reduction and favorable properties for N_2_ adsorption–activation.^15–17^ Schrauzer and co-workers conducted the pioneering systematic investigation into the photocatalytic NH_3_ synthesis using TiO_2_-based materials.^18^ In recent years, numerous studies have explored traditional metal oxides, carbonaceous materials, and layered double hydroxide semiconductors as photocatalysts for N_2_ photofixation.^19–22^ However, these semiconductor materials often exhibit wide band gaps and narrow spectral absorption characteristics, which are not conducive for efficient photon utilization in photocatalytic reactions. Inspired by the MoFe-cofactor in natural nitrogenase, molybdenum disulfide (MoS_2_) has garnered significant attention in the field of N_2_ fixation due to its structural resemblance to nitrogenase with a Mo–S configuration.^23,24^ As a two-dimensional direct bandgap semiconductor material, MoS_2_ not only possesses good photon capture ability but also can be used as a carrier to construct composite catalysts to achieve more efficient catalytic conversion. Nonetheless, MoS_2_ exhibits poor activity in photocatalytic N_2_ reduction systems using water as the electron source due to its lack of water oxidation capacity.^25^ In addition, akin to other semiconductor photocatalysts, its weak photogenerated carrier separation ability also hinders the progress of photocatalytic N_2_ reduction.^26,27^ To improve the photocatalytic activity of semiconductor photocatalysts, extensive research efforts have focused on bolstering charge separation through the construction of p–n, II-type, and S-scheme heterojunctions.^28–31^ Mimicking natural photosynthesis, the S-scheme charge transfer system has received special attention, which can simultaneously facilitate spatial carrier separation and improve redox ability.^32,33^ Additionally, monoclinic BiVO_4_ as a “star” material has a broad range of visible light utilization and excellent photoelectric stability, and has been widely used in the study of photoanodic water oxidation.^34^ Based on this, BiVO_4_ has potential to serve as a water oxidation unit for MoS_2_ to construct heterojunctions, further enhancing the efficiency of photocatalytic N_2_ fixation.

Herein, we present an innovative S-scheme heterojunction prepared by coupling sulfur vacancy enriched MoS_2_ nanosheets (V_S_-MoS_2_) with BiVO_4_ nanocrystals through electrostatic self-assembly for photocatalytic N_2_ reduction. Under simulated sunlight irradiation (100 mW cm^−2^), BiVO_4_/V_S_-MoS_2_ exhibits efficient photocatalytic N_2_ reduction activity, with a NH_3_ yield of 132.8 μmol g^−1^ h^−1^, nearly 7 times that of pure V_S_-MoS_2_ (20 μmol g^−1^ h^−1^). In addition, the experimental results from *in situ* X-ray photoelectron spectroscopy and electron spin resonance spectroscopy verify the presence of sulfur-rich vacancies on the surface of V_S_-MoS_2_ nanosheets and the successful formation of an S-scheme heterojunction, which are conducive to the improvement of N_2_ adsorption–activation and driving force of water oxidation as well as charge separation. The mechanism of the N_2_ reduction reaction is speculated using *in situ* diffuse reflectance infrared Fourier transform spectroscopy.

## 2. Experimental

### 2.1. Sample preparation

#### 2.1.1. Preparation of V_S_-MoS_2_ nanosheets

Na_2_MoO_4_·2H_2_O (243 mg) and CH_4_N_2_S (304 mg) were dissolved in ultrapure water (35 mL). After 30 min of stirring, the mixture was transferred to a 50 mL Teflon autoclave and heated at 180 °C for 48 h. Subsequently, the products were washed 3 times with a 0.1 M HCl solution to eliminate any residual nitrogen in the sample. The V_S_-MoS_2_ nanosheet powder can be obtained after vacuum drying at 60 °C for 12 h.

#### 2.1.2. Preparation of BiVO_4_ nanocrystals

In a three-neck flask, a mixture containing 484 mg of Bi(NO_3_)_3_·5H_2_O, 20 mL of octadecene, 3 mL of oleylamine, and 3 mL of oleic acid was heated to 170 °C until it became transparent, and then stored at 100 °C. NaVO_3_ (242 mg) was dissolved in ultrapure water (20 mL) at 100 °C, and added to the above three-neck flask. The resulting mixture was poured into ethanol to induce precipitation. Afterward, it was subjected to three rounds of washing with a hexane and ethanol mixture, followed by vacuum drying at 60 °C for 12 h to obtain a yellow-green BiVO_4_ nanocrystal. For further purification, the obtained BiVO_4_ quantum dots (20 mg) were dispersed in a solution comprising 10 mL of isopropanol and 1 mL of KI in DMSO (0.1 M). Ultrasonic treatment was performed for 10 min to remove the ligands introduced during the synthesis process and prevent their interference in the photocatalytic N_2_ fixation experiment.

#### 2.1.3. Preparation of BiVO_4_/V_S_-MoS_2_

V_S_-MoS_2_ nanosheets (30 mg) and BiVO_4_ nanocrystals (10 mg) were dispersed in toluene (10 mL). The mixture was ultrasonicated for 10 min and stirred for 3 h in the dark. The suspension was centrifuged followed by washing 3 times with hexane. The BiVO_4_/V_S_-MoS_2_ heterojunction can be obtained after vacuum drying at 60 °C for 12 h. In addition, a series of samples can be obtained by adjusting the mass ratio of BiVO_4_ and V_S_-MoS_2_ as 10 mg : 10 mg, 10 mg : 20 mg, 10 mg : 40 mg, denoted as BiVO_4_/V_S_-MoS_2_ (1/1), BiVO_4_/V_S_-MoS_2_ (1/2) and BiVO_4_/V_S_-MoS_2_ (1/4), respectively.

### 2.2. Photocatalytic N_2_ reduction experiment

The photocatalytic N_2_ reduction reactions were carried out in a gas–solid reaction system (25 °C). 20 mg of photocatalyst was placed on the sample table of the reactor. 200 μL of water was injected into the bottom of the reactor as the proton source. Before initiating the photocatalytic reactions, the system was heated to produce water vapor. The system was meticulously degassed to remove air with N_2_ (99.999%), where N_2_ was bubbled through 1 M HCl as well as a mixed solution of potassium permanganate and KOH to ensure the removal of potential contaminants. A 300 W xenon lamp equipped with a UVVISCUT400 filter was employed as the light source (light intensity of 100 mW cm^−2^). For detection, 2 mL of H_2_O was injected into the system to dissolve the product to form the product solution.

### 2.3. Product detection

#### 2.3.1. Detection of NH_4_^+^

The NH_4_^+^ concentration was determined with the indophenol blue method as follows: three chromogenic solutions were first prepared, divided into chromogenic agent A, chromogenic agent B, and chromogenic agent C. Chromogenic agent A was prepared by mixing NaOH (4 g), salicylic acid (5 g), and sodium citrate (5 g); chromogenic agent B was obtained by preparing a 0.05 M NaClO aqueous solution; chromogenic agent C was prepared by using 0.1 g sodium nitroferricyanide solution. The product solution (2 mL) was mixed with reagent A (2 mL), reagent B (1 mL), and reagent C (200 μL), and then left for two hours to be detected. Finally, the absorbance at a wavelength of 655 nm was measured to quantify the NH_4_^+^ content according to the established standard curve of the NH_4_Cl solution.

#### 2.3.2. Detection of NO_3_^−^

The specific process is as follows: first, prepare 1.0 ppm, 0.5 ppm, 0.2 ppm, and 0.1 ppm solutions of NaNO_3_ as the standard solutions to establish the standard curve. Then, the reaction solution (2 mL) was detected by using a model NEXION300 ion chromatograph.

#### 2.3.3. Detection of NO_2_^−^

The colorimetric method for the detection of NO_2_^−^ concentration is as follows: color reagent A was prepared by dissolving sulfonamide (0.5 g) in 50 mL of HCl solution (2 M); color reagent B was prepared by dissolving *N*-(1-naphthyl) ethylenediamine hydrochloride (20 mg) in 20 mL of ultrapure water. The reaction solution (2 mL) was mixed with color reagent A (40 μL) and color reagent B (40 μL), and then left in the dark for 10 min. The absorbance was characterized by UV-vis absorption spectroscopy, and the content of NO_2_^−^ was quantified according to the established standard curve at a wavelength of 540 nm.

#### 2.3.4. Detection of N_2_H_4_

The Watt–Chrisp method was employed to determine the concentration of hydrazine hydrate as follows: the color reagent was prepared by mixing *N*,*N*-dimethyl-4-aminobenzaldehyde (2 g), concentrated HCl (10 mL), and ethanol (100 mL). The reaction solution (2 mL) was mixed with the color reagent (2 mL), and then left in the dark for 15 min. The absorbance at ∼458 nm was obtained, and the content of N_2_H_4_ was quantified according to the established standard curve.

### 2.4. *In situ* diffuse reflectance infrared Fourier transform spectra (DRIFTS)

*In situ* diffuse reflectance infrared Fourier transform spectra were recorded using a Bruker IFS 66v Fourier transform spectrometer. The samples were mixed with KBr in a quartz mortar and then placed in an infrared reaction chamber. Pure N_2_ (99.999%) containing water vapor was continuously introduced into the experimental chamber during *in situ* characterization. The IR spectrum of pure KBr was first collected as a background spectrum. The final spectra were obtained by subtracting the background spectrum from the spectrum of the sample.

## 3. Results and discussion

### 3.1. Preparation and structure of BiVO_4_/V_S_-MoS_2_

**Scheme 1:**
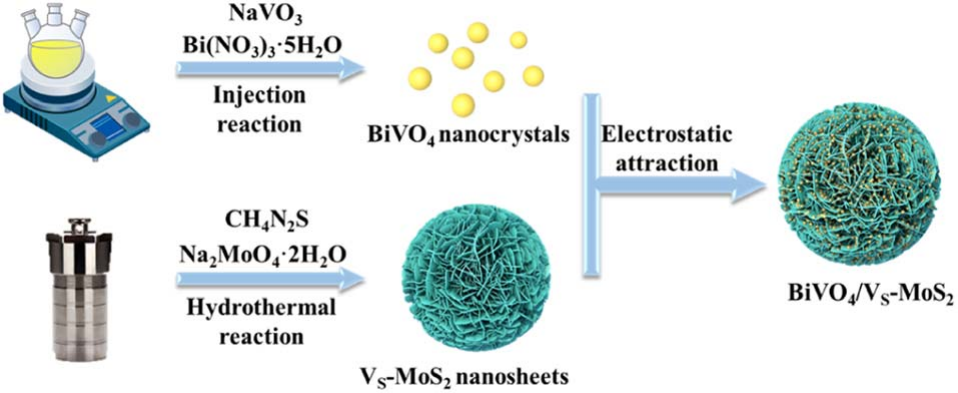
Schematic illustration of the preparation process of BiVO_4_/V_S_-MoS_2_.

**Fig. 1:**
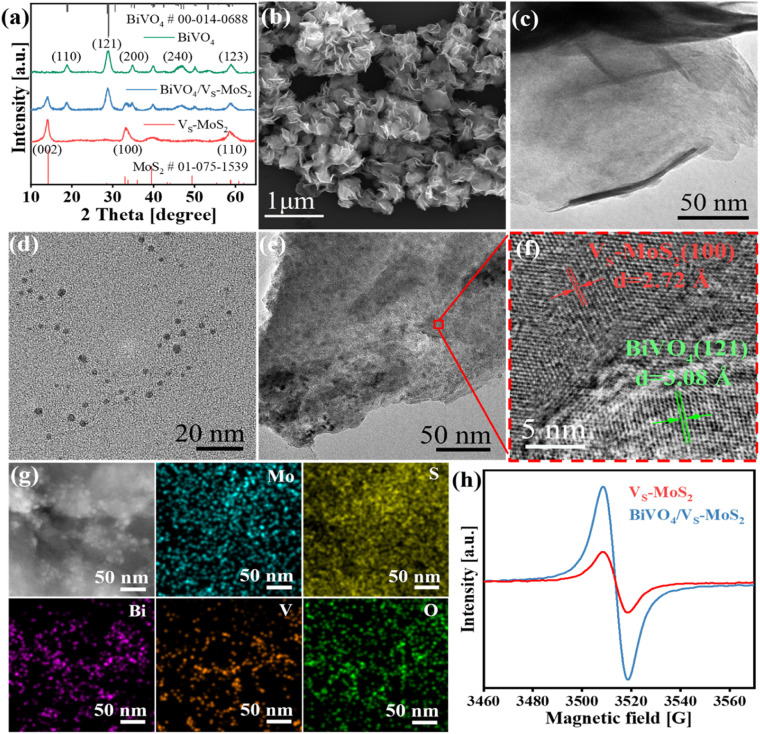
(a) XRD patterns of V_S_-MoS_2_, BiVO_4_, and BiVO_4_/V_S_-MoS_2_. (b and c) SEM and TEM images of a V_S_-MoS_2_ nanosheet. (d) TEM image of a BiVO_4_ nanocrystal. (e–g) TEM, HRTEM, and corresponding EDS images of BiVO_4_/V_S_-MoS_2_. (h) EPR spectra of V_S_-MoS_2_ and BiVO_4_/V_S_-MoS_2_.

The synthetic process of BiVO_4_/V_S_-MoS_2_ is illustrated in Scheme 1. Briefly, V_S_-MoS_2_ and BiVO_4_ were initially prepared through the hydrothermal method and thermal injection method, respectively. The measured zeta potential (*ζ*) of V_S_-MoS_2_ is negative (−45 mV), opposited with that of BiVO_4_ (33 mV) in toluene (Fig. S1†). This opposite surface charging characteristic of V_S_-MoS_2_ and BiVO_4_ should facilitate the spontaneous assembly to form BiVO_4_/V_S_-MoS_2_ in solution. X-ray diffraction (XRD) patterns (Fig. 1a) show that the as-prepared V_S_-MoS_2_ and BiVO_4_ can be indexed to the *P*63/*mmc*(194) hexagonal space group (PDF card no. 01-075-1539) and *I*2/*a*(15) monoclinic space group (PDF card no. 00-014-0688), respectively. The characteristic diffraction peaks at 14.39°, 32.8°, 39.65° and 58.56° of V_S_-MoS_2_ correspond to the (002), (100), (103), and (110) crystal planes. The diffraction peaks of BiVO_4_ are located at 18.67°, 28.95°, 35.22°, 39.78°, 46.71°, 49.96°, and 59.26°, corresponding to the (110), (121), (002), (211), (240), (−202), and (123) crystal planes. Notably, the diffraction pattern of BiVO_4_/V_S_-MoS_2_ contains the characteristic peaks of both V_S_-MoS_2_ and BiVO_4_, indicating the successful loading of BiVO_4_ nanocrystals onto V_S_-MoS_2_ nanosheets. Scanning electron microscopy (SEM) and transmission electron microscopy (TEM) measurements revealed that V_S_-MoS_2_ exhibits a nanoflower morphology (Fig. 1b), which is formed by the gathering of nanosheets (Fig. 1c). Atomic force microscopy (AFM) analysis in Fig. S2† further confirmed that V_S_-MoS_2_ has a nanosheet morphology with a mean thickness of ∼3 nm. TEM images of pure BiVO_4_ and BiVO_4_/V_S_-MoS_2_ (Fig. 1d and e) show that BiVO_4_ nanocrystals are interspersed on V_S_-MoS_2_ nanosheets, with an average size of ∼5 nm, which is almost identical to that of the pure BiVO_4_ nanocrystal (Fig. S3†). Further high-resolution TEM (HRTEM) analysis (Fig. 1f) showed clear lattice fringes of 2.72 Å and 3.08 Å, corresponding to the (100) plane of V_S_-MoS_2_ (ref. 35) and the (121) plane of monoclinic BiVO_4_ (ref. 36) (Fig. S4†), respectively, confirming the presence of BiVO_4_ nanocrystals on the surface of V_S_-MoS_2_ nanosheets. The corresponding energy-dispersive X-ray spectroscopy (EDS) mapping analysis (Fig. 1g) demonstrated the homogeneous distribution of Mo, S, Bi, V, and O elements on BiVO_4_/V_S_-MoS_2_, further verifying the construction of BiVO_4_/V_S_-MoS_2_.

To gain insights into the structural characteristics of the as-prepared photocatalysts, electron paramagnetic resonance (EPR) measurements were performed initially. As depicted in Fig. 1h, a distinct EPR signal at *g* = 2.003 can be recognized for V_S_-MoS_2_, signifying the formation of the sulfur vacancies on the surface of V_S_-MoS_2_.^37^ In the case of BiVO_4_/V_S_-MoS_2_, a stronger EPR signal is detected, providing evidence for the formation of heterojunction interfaces, which can turn spin-coupled electron pairs into unpaired electron states.^38^ Sulfur vacancies can serve as active sites to promote carrier capture and N_2_ adsorption and activation.^39^ The X-ray photoelectron spectra (XPS) were further analyzed to investigate the potential interfacial interaction and charge transfer processes within the BiVO_4_/V_S_-MoS_2_ heterojunction. Doublet XPS characteristic peaks can be identified at 229.3 eV and 232.4 eV in pure V_S_-MoS_2_, attributed to Mo 3d_5/2_ and Mo 3d_3/2_ (Fig. S5a†), which can confirm the reduction of Mo^6+^ to Mo^4+^ during the growth of V_S_-MoS_2_ nanosheets.^40^ In addition, a comparison between pure V_S_-MoS_2_, BiVO_4_, and BiVO_4_/V_S_-MoS_2_ reveals noticeable shifts in binding energy for Mo 3d, S 2p, Bi 4f, and V 2p. Specifically, Mo 3d and S 2p exhibit positive shifts of 0.50 eV and 0.20 eV, while Bi 4f and V 2p display negative shifts of 0.20 eV and 0.20 eV, respectively (Fig. S5b–d†). These charge redistributions within the BiVO_4_/V_S_-MoS_2_ heterojunction reflect a strong chemical interaction at the interface of the BiVO_4_/V_S_-MoS_2_ heterojunction. This robust interfacial electronic coupling in BiVO_4_/V_S_-MoS_2_ is expected to facilitate the charge transfer between the V_S_-MoS_2_ nanosheets and BiVO_4_ nanocrystals.

### 3.2. S-scheme mechanism of the BiVO_4_/V_S_-MoS_2_ heterojunction

**Fig. 2:**
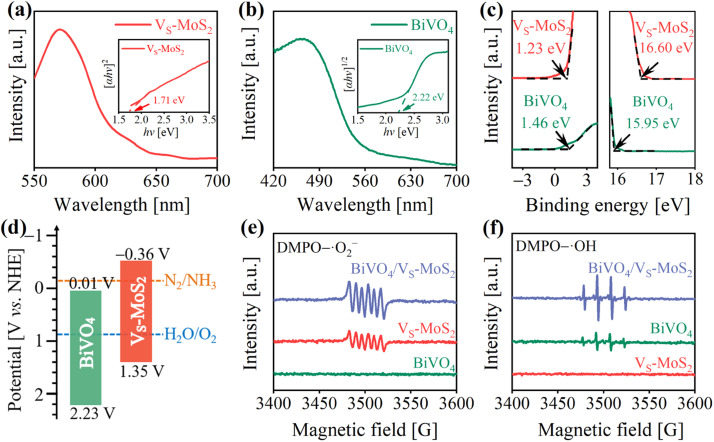
(a and b) UV-vis DRS spectra of V_S_-MoS_2_ and BiVO_4_. The insets show the corresponding Tauc plots. (c) UPS spectra of V_S_-MoS_2_ and BiVO_4_. (d) Energy band structures of V_S_-MoS_2_ and BiVO_4_ (pH = 7). EPR signals of (e) DMPO–·O_2_^−^ and (f) DMPO–·OH over V_S_-MoS_2_, BiVO_4_, and BiVO_4_/V_S_-MoS_2_ under illumination.

The energy levels and interfacial charge transfer processes of BiVO_4_/V_S_-MoS_2_ were explored to evaluate the photocatalytic N_2_ reduction potential of the as-prepared photocatalysts. Fig. 2a and b show the UV-vis diffuse reflectance spectra (DRS), revealing a good light response for both the V_S_-MoS_2_ nanosheets and BiVO_4_ nanocrystals. From the corresponding Tauc plots (insets of Fig. 2a and b), the band gaps (*E*_g_) of V_S_-MoS_2_ and BiVO_4_ can be deduced to be 1.71 eV and 2.22 eV, respectively. To ascertain the energy band structures of V_S_-MoS_2_ and BiVO_4_, the onset edge (*E*_i_) and the secondary electron cutoff (*E*_cutoff_) were determined by carrying out ultraviolet photoelectron spectroscopy (UPS) (Fig. 2c). According to the equation *E*_VB_ = 21.22 − (*E*_cutoff_ − *E*_i_), the valence band edge potentials (*E*_VB_) can be calculated to be 1.35 V and 2.23 V *versus* the standard hydrogen electrode (*vs.* NHE) for V_S_-MoS_2_ and BiVO_4_, respectively. By combining the values from *E*_g_ and *E*_VB_ analysis, the corresponding conduction band edge potentials (*E*_CB_) can be calculated to be −0.36 V and 0.01 V (*vs.* NHE) for V_S_-MoS_2_ and BiVO_4_, respectively. The resultant energy band structures of V_S_-MoS_2_ and BiVO_4_ are presented in Fig. 2d. The photogenerated electrons in V_S_-MoS_2_ possess sufficient energy to drive the photoreduction of N_2_ to NH_3_ (−0.13 V *vs.* NHE),^41^ while their driving force for H_2_O oxidation is relatively weak. Meanwhile, the photogenerated holes in BiVO_4_ can trigger H_2_O photooxidation to O_2_ (0.82 V *vs.* NHE, pH = 7), while they are insufficient for the photoreduction of N_2_. In addition, the Fermi levels were also estimated according to the UPS spectra, to be −4.62 eV and −5.27 eV (*vs.* vacuum) for V_S_-MoS_2_ and BiVO_4_, respectively. These results reveal that both V_S_-MoS_2_ and BiVO_4_ are n-type semiconductors, consistent with the results from the Mott–Schottky tests, where the slopes of Mott–Schottky curves for both BiVO_4_ and V_S_-MoS_2_ are positive (Fig. S6†). From the above results and analysis, we can preliminarily deduce that, given the higher Fermi level of V_S_-MoS_2_ compared to that of BiVO_4_, the free electrons in V_S_-MoS_2_ would spontaneously flow to BiVO_4_ to establish a Fermi level equilibrium upon contact (Fig. S7(I–II)†). A built-in electric field and band bending of the interface in BiVO_4_/V_S_-MoS_2_ would be formed. Driven by the interfacial built-in electric field, photogenerated electrons in BiVO_4_ would recombine with the photogenerated holes in V_S_-MoS_2_ (Fig. S7(III)†), facilitating the formation of the S-scheme charge transfer pathway.

To scrutinize the S-scheme charge transfer pathway in the BiVO_4_/V_S_-MoS_2_ heterojunction, *in situ* irradiated XPS (ISI-XPS) spectra were first measured to detect photoinduced changes in the electron cloud density around the nuclei of elements. As depicted in Fig. S8a and b,† the binding energies of Mo 3d and V 2p in BiVO_4_/V_S_-MoS_2_ demonstrate a negative displacement (∼0.40 eV) and positive movement (∼0.30 eV) under illumination, respectively, compared with those in the dark. These binding energy changes indicate the accumulation of photogenerated electrons on V_S_-MoS_2_ and photogenerated holes on BiVO_4_, revealing the occurrence of S-scheme interfacial charge transfer within the BiVO_4_/V_S_-MoS_2_ heterojunction. To further confirm this S-scheme interfacial charge transfer mechanism in the BiVO_4_/V_S_-MoS_2_ heterojunction, the EPR spectra were recorded using 5,5-dimethyl-1-pyrroline *N*-oxide (DMPO) as the trapping agent.^42^ In the case of pure V_S_-MoS_2_, only the DMPO–·O_2_^−^ characteristic signal can be identified (Fig. 2e), as its *E*_VB_ potential is insufficient to drive the oxidation of H_2_O to ·OH. Conversely, in pure BiVO_4_, only the DMPO–·OH characteristic signal can be recognized (Fig. 2f), due to the insufficient driving force of photogenerated electrons in *E*_CB_ to trigger the reduction of O_2_ to ·O_2_^−^ (−0.33 V *vs.* NHE). For the BiVO_4_/V_S_-MoS_2_ heterojunction, more obvious DMPO–·O_2_^−^ and DMPO–·OH signals can be simultaneously identified, providing compelling evidence for the establishment of an interfacial S-scheme charge transport channel. That is to say, the photogenerated electrons in BiVO_4_ and the photogenerated holes in V_S_-MoS_2_ initially recombine through the interfacial S-scheme channel, leading to the accumulation of photogenerated holes on BiVO_4_ and photogenerated electrons on V_S_-MoS_2_. This spatial separation of charge carriers in the BiVO_4_/V_S_-MoS_2_ heterojunction would more effectively promote their participation in the redox reaction.

### 3.3. Photogenerated carrier evolution

**Fig. 3:**
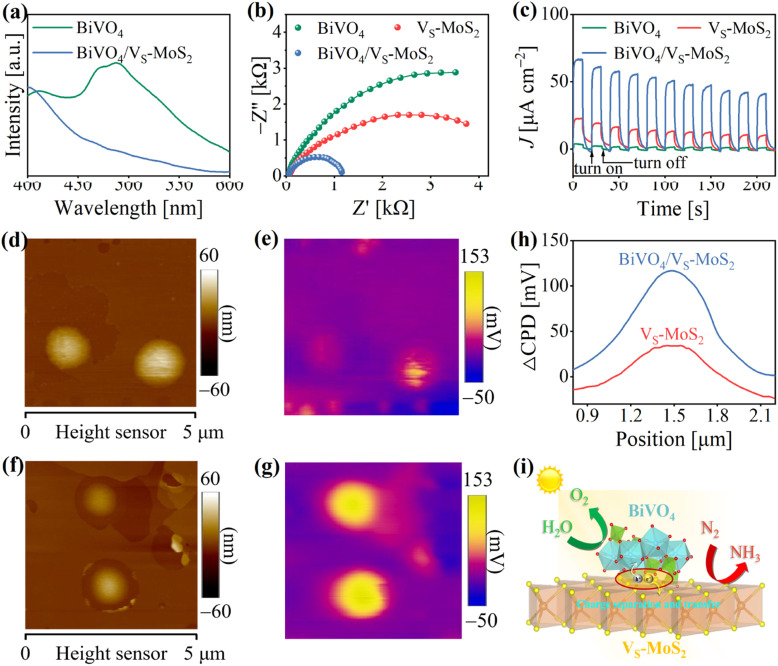
(a) Steady-state photoluminescence spectra of BiVO_4_ and BiVO_4_/V_S_-MoS_2_. (b and c) EIS plots and *I*–*t* curves of BiVO_4_, V_S_-MoS_2_, and BiVO_4_/V_S_-MoS_2_ plotted at a bias potential of −0.4 V *vs.* Ag/AgCl. AFM height images of (d) V_S_-MoS_2_ and (f) BiVO_4_/V_S_-MoS_2_. SPV images of (e) V_S_-MoS_2_ and (g) BiVO_4_/V_S_-MoS_2_ under illumination. (h) Potential difference changes (ΔCPD) of V_S_-MoS_2_ and BiVO_4_/V_S_-MoS_2_ by subtracting the potential under dark conditions from that under illumination. (i) Schematic illustration of the photogenerated carrier separation.

In general, the evolution of photogenerated charges within photocatalysts plays a pivotal role in determining their photocatalytic activity.^43,44^ To evaluate the behavior of photogenerated carriers in BiVO_4_/V_S_-MoS_2_, steady-state photoluminescence (PL) spectrum measurements were first performed. As presented in Fig. 3a, the PL spectrum of BiVO_4_ displays an intrinsic emission band at ∼490 nm under 365 nm excitation, which is related to photogenerated electron–hole recombination. Upon the construction of the BiVO_4_/V_S_-MoS_2_ heterojunction, the PL spectrum exhibits significant quenching, indicating the effective transfer of photogenerated carriers between the BiVO_4_ nanocrystals and V_S_-MoS_2_ nanosheets. In addition, the photoelectrochemical properties of the samples were characterized to assess the potential positive influence of the construction of a heterostructure on charge evolution behavior. Notably, under light irradiation, BiVO_4_/V_S_-MoS_2_ exhibits a smaller charge-transport resistance compared with both pure BiVO_4_ and V_S_-MoS_2_ (Fig. 3b and Table S1†), indicating that the construction of the electric field in the heterojunction improves the charge transport characteristics. From the transient photocurrent (*I*–*t*) measurements (Fig. 3c), it is evident that BiVO_4_/V_S_-MoS_2_ has a higher current density than both BiVO_4_ and V_S_-MoS_2_, providing further evidence of expedited charge separation kinetics within the BiVO_4_/V_S_-MoS_2_ heterojunction. Moreover, to more intuitively reveal the space charge separation, Kelvin probe force microscopy (KPFM) was utilized to measure the surface photovoltage (SPV) response of photocatalysts under light. Atomic force microscope (AFM) images (Fig. 3d and f) present the morphology outline of V_S_-MoS_2_ and BiVO_4_/V_S_-MoS_2_ nanosheet clusters, which are basically consistent with the SEM results (Fig. 1b). Under illumination, the SPV response zone (Fig. 3e and g) emerges, aligning with the morphology outlines of the samples. These observations also indicate the spatial charge separation and redistribution after light irradiation.^45^ As shown in Fig. 3h, pure V_S_-MoS_2_ presents a feeble SPV signal under light irradiation, primarily due to rapid photogenerated carrier recombination. Benefiting from the construction of heterojunctions, BiVO_4_/V_S_-MoS_2_ displays a noticeable enhancement of the SPV response, of ∼100 mV, far surpassing that of pure V_S_-MoS_2_. Ultimately, these characterization studies underscore that the construction of heterojunctions can promote charge separation, and enhance the accumulation of electrons/holes on V_S_-MoS_2_ and BiVO_4_, respectively, which is beneficial for these carriers to participate in the subsequent multi-electron transfer processes during the photoreduction N_2_ reaction (Fig. 3i).

### 3.4. Photocatalytic N_2_ reduction activity

**Fig. 4:**
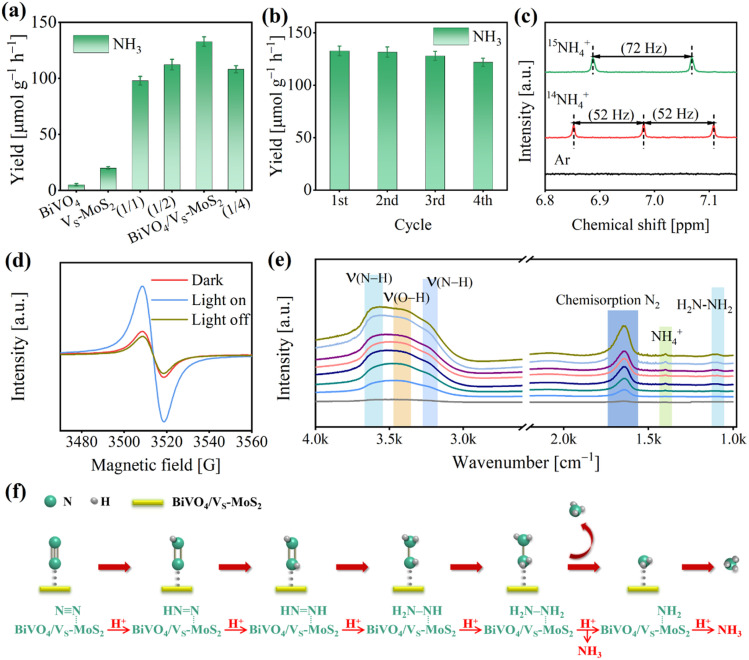
(a) Photocatalytic NH_3_ production rates for BiVO_4_, V_S_-MoS_2_, BiVO_4_/V_S_-MoS_2_, BiVO_4_/V_S_-MoS_2_ (1/1), BiVO_4_/V_S_-MoS_2_ (1/2), and BiVO_4_/V_S_-MoS_2_ (1/4) under 4 h light irradiation. (b) NH_3_ synthesis for four cyclic tests of BiVO_4_/V_S_-MoS_2_. (c) The ^1^H NMR spectra of products with ^14^N_2_ or ^15^N_2_ as the reaction atmosphere. (d) *In situ* EPR spectra of BiVO_4_/V_S_-MoS_2_ in the dark or under light irradiation in a N_2_ atmosphere or light off in a N_2_ atmosphere. (e) *In situ* DRIFTS spectra of the photoreduction N_2_ reaction. (f) The proposed reaction pathway of the photoreduction N_2_ reaction.

The photocatalytic N_2_ reduction reactions were conducted in a gas–solid reaction apparatus (Fig. S9†) containing N_2_ and H_2_O vapor under simulated sunlight irradiation (100 mW cm^−2^). To ensure the accuracy of our experiments, the samples were pretreated with a DMSO solution of KI. This step is crucial to remove any residual ligands introduced during the synthesis process, preventing any interference with the experimental results (Fig. S10,† details are described in the Experimental section). The photocatalytic activities were evaluated by evaluating the NH_4_^+^ production using the indophenol blue method (Fig. S11,† details are described in the Experimental section).^46^ As illustrated in Fig. S12,† a prominent absorption signal at ∼655 nm can be observed, indicating that N_2_ is effectively photoreduced to NH_3_ in the presence of light for BiVO_4_/V_S_-MoS_2_. The NH_3_ generation performances for the as-prepared photocatalysts are presented in Fig. 4a and S13.† For pure BiVO_4_, there is virtually no NH_3_ synthesis activity due to the insufficient driving force of electron energy in its conduction band (Fig. 2d). Meanwhile, V_S_-MoS_2_ exhibits a weak N_2_ photoreduction to NH_3_ activity, with a rate of 20.0 ± 1.1 μmol g^−1^ h^−1^, resulting from its insufficient thermodynamic driving force for H_2_O oxidation and serious charge recombination. Upon combining the V_S_-MoS_2_ nanosheets with the BiVO_4_ nanocrystals, the corresponding photocatalytic N_2_ reduction activity improves significantly, reaching 132.8 ± 4.2 μmol g^−1^ h^−1^ within 4 hours, which is nearly 7-fold that of pure V_S_-MoS_2_. The apparent quantum efficiency at 575 nm can reach 0.3% (Fig. S14†). Certainly, we also analyzed the NH_4_^+^ production by ion chromatography (Fig. S15†), and the results are in agreement with those obtained by the indophenol blue method. Obviously, this improvement of NH_3_ evolution activity can be attributed to the increased driving force for water oxidation and the enhanced separation of photogenerated carriers in the BiVO_4_/V_S_-MoS_2_ heterojunction, compared with pure V_S_-MoS_2_ nanosheets. By adjusting the mass ratio of BiVO_4_ and V_S_-MoS_2_ (Fig. S16†), the NH_3_ evolution activities of the samples were screened, in which BiVO_4_/V_S_-MoS_2_ with a mass ratio (BiVO_4_ : V_S_-MoS_2_) of 1 : 3 exhibits the best performance (Fig. 4a).

Moreover, the O_2_ evolution activity of BiVO_4_/V_S_-MoS_2_ was also measured through a gas chromatograph, to be 110.0 ± 4.5 μmol g^−1^ h^−1^ (Fig. S17†). This result suggests that the rate of consumed electrons (*R*_electron_) for N_2_ reduction to NH_3_ is nearly equal to the rate of consumed holes (*R*_hole_) for water oxidation to O_2_, in accordance with the formulae of *R*_elecreon_ = 3 × *R*_NH_3__ and *R*_hole_ = 4 × *R*_O_2__. This balance signifies the effective utilization of the photogenerated electrons and holes. Based on the above analysis, the whole photocatalytic reaction including N_2_ reduction and H_2_O oxidation by BiVO_4_/V_S_-MoS_2_ can be described as 2N_2_ + 6H_2_O → 4NH_3_ + 3O_2_. Additionally, the robustness of BiVO_4_/V_S_-MoS_2_ was evaluated by reemploying the photocatalyst in four cycles of tests (Fig. 4b), which reveals little decrement in activity. In addition, the XRD patterns (Fig. S18†) and TEM images (Fig. S19†) of BiVO_4_/V_S_-MoS_2_ after the photocatalytic reaction exhibit no obvious changes, confirming the good stability of the photocatalyst in this gas–solid photocatalytic system. The Mo 3d and Bi 4f XPS spectra of BiVO_4_/V_S_-MoS_2_ after catalysis show almost no significant changes, further suggesting the maintenance of the local coordination environment of the Bi and Mo sites.

To elucidate the origin of NH_3_ and O_2_ during the photocatalytic reaction, several controlled experiments were carried out using BiVO_4_/V_S_-MoS_2_ as the photocatalyst (Fig. S21†). In the absence of a photocatalyst (BiVO_4_/V_S_-MoS_2_), N_2_, light, or H_2_O, there is nearly no NH_3_ evolution, unequivocally demonstrating that the N_2_ reduction reaction is indeed a photocatalytic reaction using BiVO_4_/V_S_-MoS_2_ as the photocatalyst with H_2_O as the proton source. According to the characterization by the colorimetric method and ion chromatography (the details are described in the Experimental section), nearly no NO_2_^−^ and NO_3_^−^ signals in the product system can be detected before and after the photocatalytic reaction (Fig. S22 and S23†). In addition, we have further measured the N content in the obtained photocatalyst by EDS analysis (Fig. S24 and Table S2†), and no N atoms are present. These results confirm that NH_3_ remained unoxidized, and substantiate that NH_3_ originated solely from N_2_ reduction, excluding impurity conversion as a source. To further verify the origin of NH_3_ in the product system, isotope labeling experiments were performed using BiVO_4_/V_S_-MoS_2_ as the photocatalyst in atmospheres containing ^15^N_2_, ^14^N_2_, or Ar, respectively (Fig. S25†). More specifically, no NH_4_^+^ signal is detected under a Ar atmosphere in the nuclear magnetic resonance (NMR) spectra, while distinct signals belonging to ^15^NH_4_^+^ or ^14^NH_4_^+^ can be observed in a ^15^N_2_ or ^14^N_2_ atmosphere, respectively (Fig. 4c). These results directly confirm that NH_4_^+^ is indeed generated from the N_2_ in the system. In addition, the mass spectrometry analysis of the product (Fig. S26†) was also performed, revealing an ^18^O_2_ signal at *m*/*z* = 36, which confirms that O_2_ is produced from H_2_O oxidation.

To gain insight into the photocatalytic N_2_ reduction process of the BiVO_4_/V_S_-MoS_2_ heterojunction, we conducted further characterization to explore the active sites and monitor the evolution of intermediates during the photocatalytic reaction, by using *in situ* EPR spectra and *in situ* diffuse reflectance infrared Fourier transform spectra (DRIFTS), respectively. As presented in Fig. 4d, the intensity of the sulfur vacancy signal for BiVO_4_/V_S_-MoS_2_ exhibits an increment when exposed to light irradiation for 15 min, and decreases once the light was turned off for 10 min, suggesting that the sulfur vacancies facilitate the trapping of photogenerated electrons from the conduction band.^47,48^ The exposed Mo site at the sulfur vacancy is in a coordination unsaturated state, and can act as the photocatalytic active site bonding with N_2_. The trapped photogenerated electrons at sulfur vacancies can transfer to N_2_ molecules to achieve N_2_ reduction and transformation. As the photocatalytic N_2_ reduction reaction proceeded, a series of absorption signals were detected belonging to intermediates adsorbed on BiVO_4_/V_S_-MoS_2_ (Fig. 4e). Specifically, an obvious peak at 1648 cm^−1^ belonging to the chemisorbed N_2_ (–N≡N) appeared,^49^ which can be attributed to the efficient adsorption and activation of N_2_ by the Mo sites at the vacancies of the nanosheets.^50^ The peak at 3432 cm^−1^ is attributed to *ν*(O–H) of H_2_O, which serves as the proton source for N_2_ activation. The overlapped signal bands at 3230 cm^−1^ and 3555 cm^−1^ can be attributed to *ν*(N–H) stretching modes of NH_3_, and the peak at 1403 cm^−1^ can be attributed to NH_4_^+^ adsorption. The enhancement of these characteristic signals implies the progression of N_2_ activation and conversion to NH_3_*via* a multistep proton-coupled electron transfer (PCET) process over BiVO_4_/V_S_-MoS_2_. In addition, a peak at 1102 cm^−1^ can be identified, originating from the H_2_N–NH_2_ characteristic signal.^51^ Given that there is no hydrazine formation in the product (Fig. S27†), we thus speculate that the photocatalytic N_2_ fixation process on BiVO_4_/V_S_-MoS_2_ follows an associative alternating pathway (Fig. 4f). In this pathway, hydrogenation occurs alternatively on two N atoms, with the final step involving the cleavage of the N–N bond to generate the first NH_3_ molecule, followed by the last hydrogenation and another NH_3_ desorption.

## 4. Conclusions

In summary, we have successfully prepared an S-scheme heterojunction of BiVO_4_/V_S_-MoS_2_ by coupling the N_2_-activated component (V_S_-MoS_2_) and the water-oxidized module (BiVO_4_) to facilitate the process of photocatalytic N_2_ reduction. BiVO_4_/V_S_-MoS_2_ exhibits efficient NH_3_ synthesis activity using H_2_O as a proton source under illumination (100 mW cm^−2^), with an NH_3_ yield of 132.8 μmol g^−1^ h^−1^, nearly 7 times higher than that of pure V_S_-MoS_2_. Our experimental findings underscore the pivotal roles played by the V_S_-MoS_2_ nanosheet with sulfur-rich vacancies in enhancing N_2_ adsorption and activation. Furthermore, the construction of the S-scheme heterojunction substantially elevates the driving force for H_2_O oxidation and significantly improves charge separation within the system. Moreover, an alternate association pathway for N_2_ photoreduction reaction is proposed according to the *in situ* DRIFTS characterization. This study presents an efficient example for achieving the N_2_ conversion process under mild conditions.

## Author contributions

Han-Ying Luo: data curation, formal analysis, validation. Zhao-Lei Liu: data curation, formal analysis, validation. Meng-Ran Zhang: formal analysis, supervision. Yan-Fei Mu: validation, writing – original draft preparation, funding acquisition. Min Zhang: conceptualization, funding acquisition, supervision, validation, writing – reviewing and editing.

## Conflicts of interest

There are no conflicts of interest to declare.

## Supplementary Material

NA-006-D3NA01091K-s001
