# Supplementary material for: Construction of a BiVO4/VS-MoS2 S-scheme heterojunction for efficient photocatalytic nitrogen fixation
Source: Nanoscale Adv. 2024 Feb 20;6(6):1781–9. doi: 10.1039/d3na01091k (PMC10929607; doi:10.1039/d3na01091k)
Supplement: NA-006-D3NA01091K-s001 [file NA-006-D3NA01091K-s001.pdf]

Electronic Supplementary Material (ESI) for Nanoscale Advances.
This journal is © The Royal Society of Chemistry 2024

Electronic supplementary information

Construction of $BiVO_4/V_S$-MoS_2 S-scheme heterojunction for efficient photocatalytic nitrogen fixation

Han-Ying Luo,‡[a] Zhao-Lei Liu,‡[a] Meng-Ran Zhang,[a] Yan-Fei Mu,*[b] Min Zhang*[a]

[a]MOE International Joint Laboratory of Materials Microstructure, Institute for New Energy Materials and Low Carbon Technologies, School of Materials Science and Engineering, School of Chemistry and Chemical Engineering, Tianjin University of Technology, Tianjin 300384, China.

[b]School of Chemistry and Chemical Engineering, Yangzhou University, Yangzhou, 225009, Jiangsu, China.

‡These authors contributed equally to this work.

*Corresponding author.

E-mail: 007916@yzu.edu.cn; zm2016@email.tjut.edu.cn.

1. Experimental section

Materials: Bismuth nitrate pentahydrate ($Bi(NO_3)_3 \cdot 5H_2O$, 99%), sodium molybdate dihydrate ($Na_2MoO_4 \cdot 2H_2O$, 99.9%), thiourea (CH_4N_2S, AR, 99%), dimethyl sulfoxide (DMSO, HPLC, ≥99.9%), isopropanol (HPLC, ≥99.5%), sodium metavanadate ($NaVO_3$, 99%), potassium bromide (KI, HPLC), tetrabutylammonium hexafluorophosphate ($TBAPF_6$), sodium hydroxide (NaOH, 99%), anhydrous ethanol (99.9%), and toluene (99.5%) were purchased from china pharmaceutical group co. ltd. $^{15}N_2$ (≥99%) was purchased from wuhan isotope technology co. ltd. $H_2{}^{18}O$ (≥98%) was purchased from Energy Chemical. Unless otherwise specified, all chemicals are commercially available and used without further purification.

Instrument: The SEM images were obtained from FEI Verios 460L. The TEM images were obtained from LaB6 Gun (Tecnai G2 Spirit TWIN, FEI, USA). The HRTEM and STEM−EDS images were obtained from Talos F200X transmission electron microscope (FEI). The PXRD patterns were collected from a Rigaku diffractometer (SmartLab, 9 kW, Rigaku, Japan). The FTIR spectra were obtained from the PerkinElmer Frontier mid–IR FTIR spectrometer. The XPS measurements were performed on an ESCALAB Model 250Xi X−ray photoelectron spectrometer. The UPS spectra were obtained from an ESCALAB250Xi type X−ray photoelectron spectrometer (Thermo scientific). The ion chromatograms were collected from an ECO ion chromatograph (NEXION300, PerkinElmer, America). The EPR spectra were obtained from an EPR spectrometer (EXMplus6−1, Bruker, Germany). The ^{15}N NMR spectra of the reaction solutions were determined using a Bruker NMR spectrometer (A V ANCE III HD 400 MHz). The UV−Visible spectra were obtained from a Lambda 750 UV/VIS/NIR by PerkinElmer. The Zeta potentials were characterized using a 90Plus Zeta by BIC, USA. The atomic force microscope height mapping images and surface photovoltage response measurements were detected through an *in-situ* photo-assisted Kelvin probe force microscopy (KPFM) technique on a Bruker Dimension Fast Scan AFM system with the model of conductive probe SCM-PIT-V2.

Photoelectrochemical experiments: All photoelectrochemical characterizations were performed on a CHI 760E electrochemical workstation with a three−electrode configuration with catalyst attached FTO glass (0.5 cm^2), Pt sheet, and Ag/AgCl (3 M KCl) as the working, counting, and reference electrodes, respectively. An acetonitrile-filled cell with 0.1 M $TBAPF_6$ was used as the electrolyte. The light source and optical density were consistent with the photocatalytic N_2 reduction test.

2. Supplementary Figs S1−S26 and Table S2

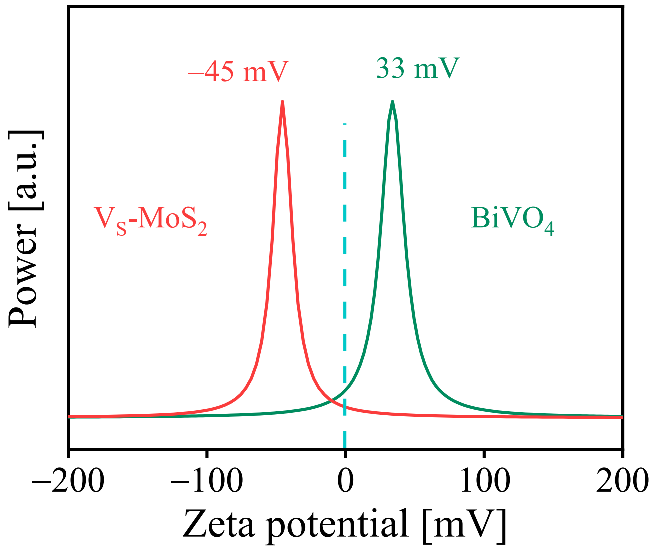

Fig. S1. Zeta potentials of as-prepared V_S-MoS_2 nanosheet and $BiVO_4$ nanocrystal.

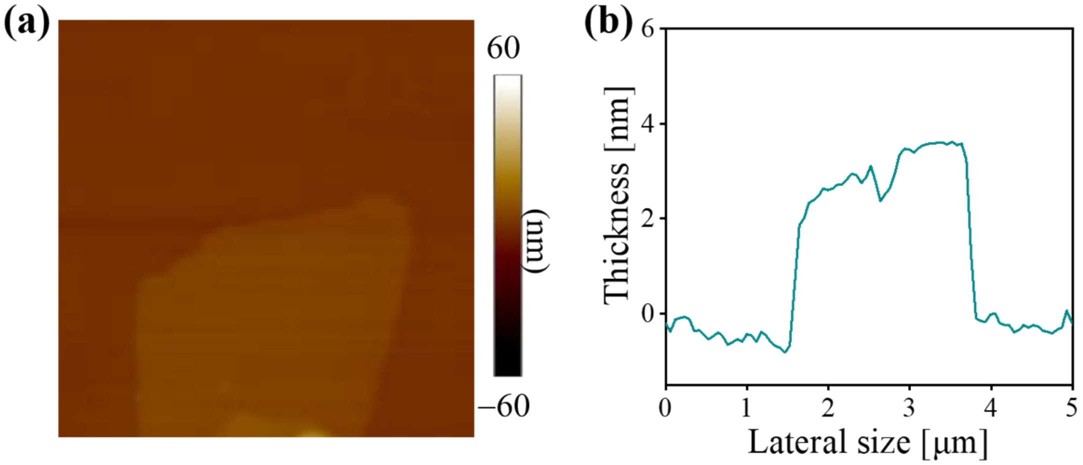

Fig. S2. (a) AFM image and (b) the corresponding height cross-sectional profile of V_S-MoS_2 deposited on the silicon substrate.

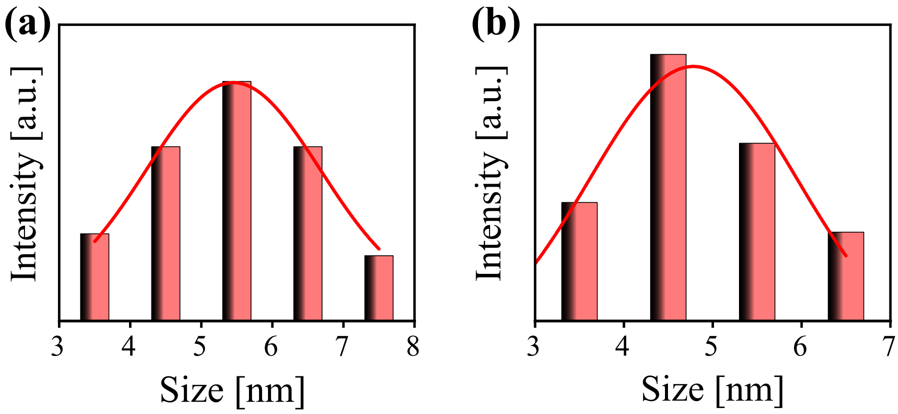

Fig. S3. The size distributions of (a) pristine $BiVO_4$ and (b) $BiVO_4$ in $BiVO_4/V_S$-MoS_2 heterojunction.

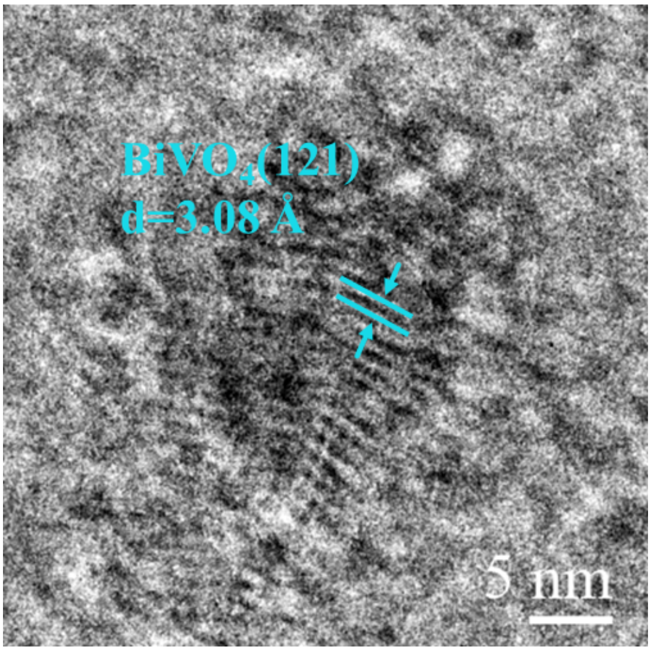

Fig. S4. HRTEM image of as-prepared $BiVO_4$ nanocrystal.

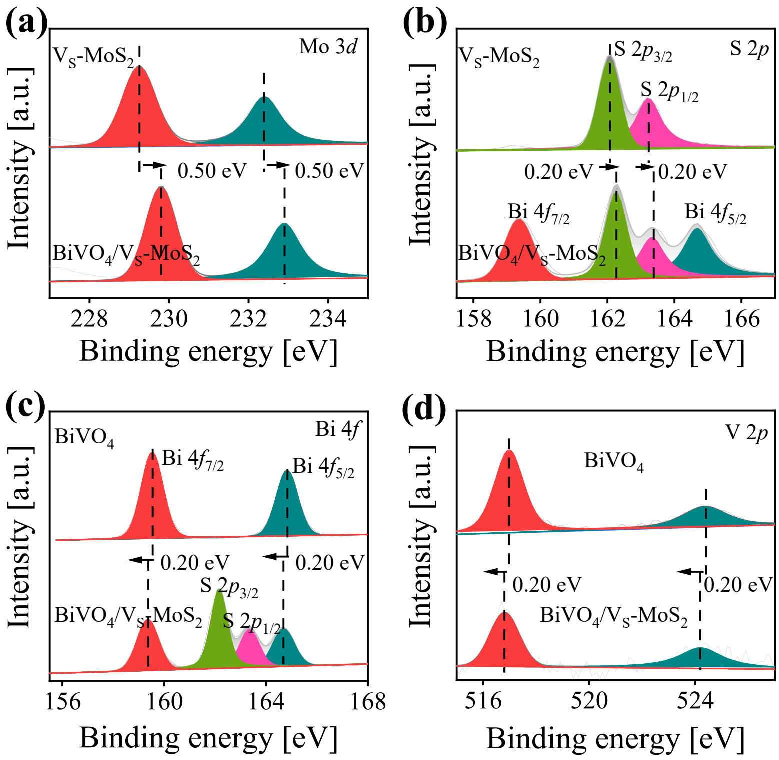

Fig. S5. High-resolution XPS spectra of V_S-MoS_2, $BiVO_4$ and $BiVO_4$/V_S-MoS_2, (a) Mo 3*d*, (b) S 2*p*, (c) Bi 4*f*, and (d) V 2*p*.

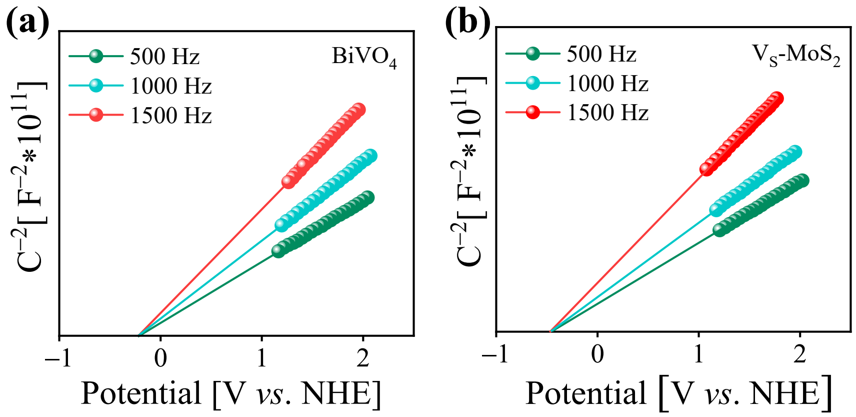

Fig. S6. Mott−Schottky plots of (a) $BiVO_4$ and (b) V_S-MoS_2.

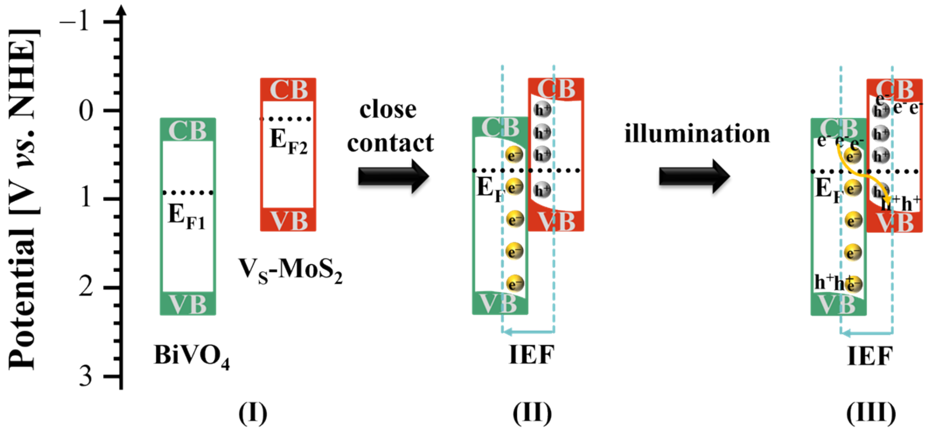

Fig. S7. The formation and working mechanism of S-scheme in $BiVO_4$/V_S-MoS_2: (I) energy band structures of $BiVO_4$ and V_S-MoS_2 before contact; (II) Fermi level alignment and formation of the built-in electric field; (III) charge transfer process under illumination. (E_{F1}: Fermi level of $BiVO_4$, E_{F2}: Fermi level of V_S-MoS_2, E_F: Fermi level of $BiVO_4$/V_S-MoS_2, IEF: built-in electric field.)

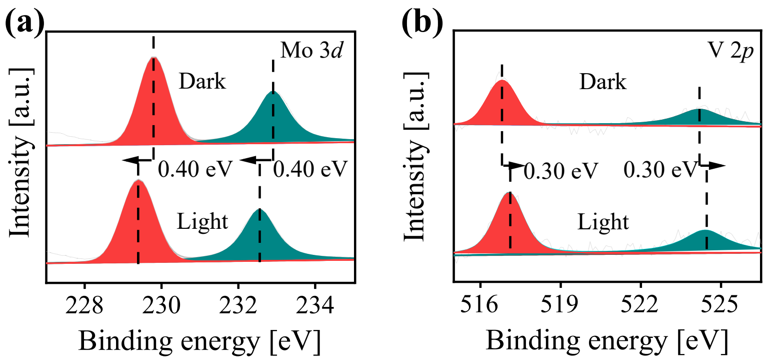

Fig. S8. High-resolution XPS spectra of $BiVO_4/V_S$-MoS_2 in the dark or under 300 W Xe lamp irradiation. (a) Mo 3*d* and (b) V 2*p*.

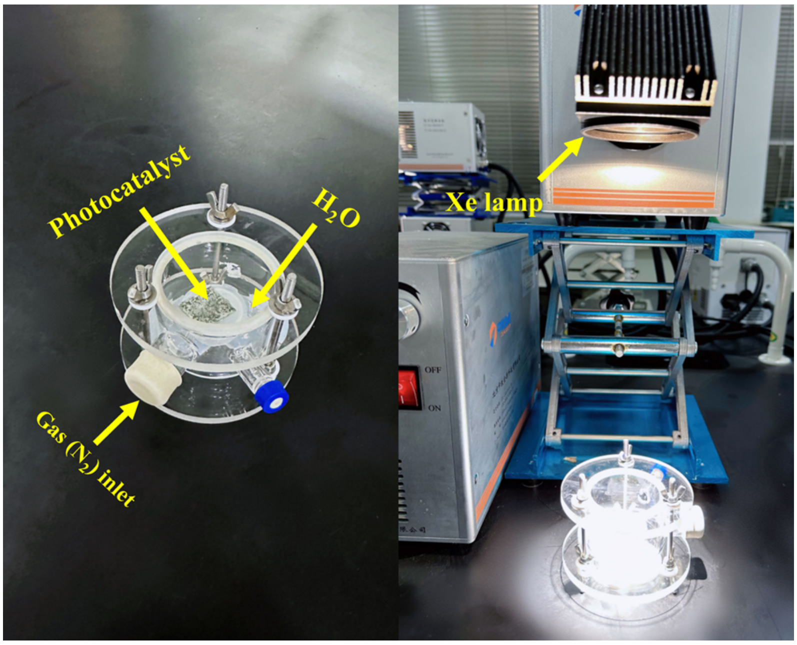

Fig. S9. The gas−solid reaction apparatus for photocatalytic N_2 reduction.

Table S1. Fitting parameters of EIS spectra in Fig. 3b.

Sample	R_1 [Ω]	R_2 [Ω]
$BiVO_4$	76	7000
V_S-MoS_2	47	4000
$BiVO_4$/V_S-MoS_2	30	1214

R_1: series resistance.

R_2: charge transport resistance.

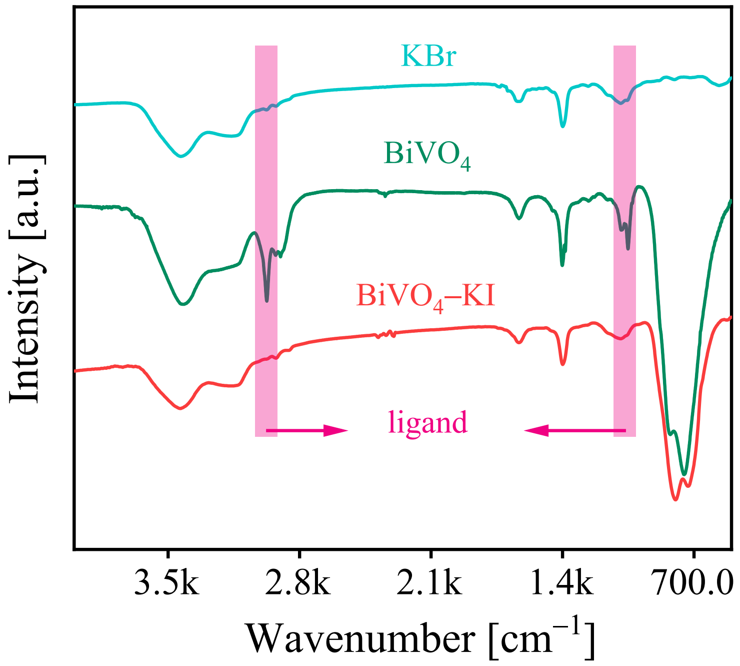

Fig. S10. FT–IR spectra of $BiVO_4$ and $BiVO_4$–KI.

The $BiVO_4$ nanocrystal was first synthesized by the thermal injection method. Peaks at 1052 cm^{-1} and 2978 cm^{-1} can be assigned to the characteristic vibration of the ligands. It can be seen that the ligands have been completely removed after treatment with DMSO solution of KI.

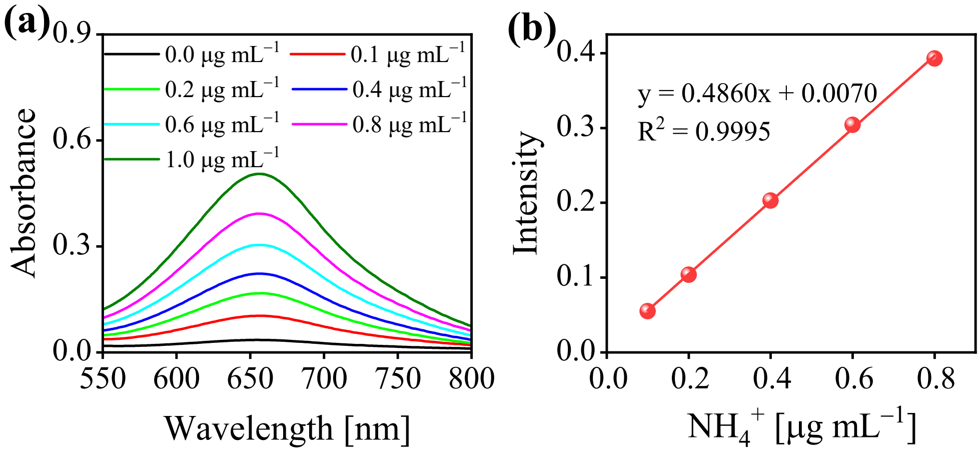

Fig. S11. (a) UV−Vis absorption spectra of NH_4^+ solutions with different concentrations measured by the indophenol blue method. (b) The standard curve of NH_4^+.

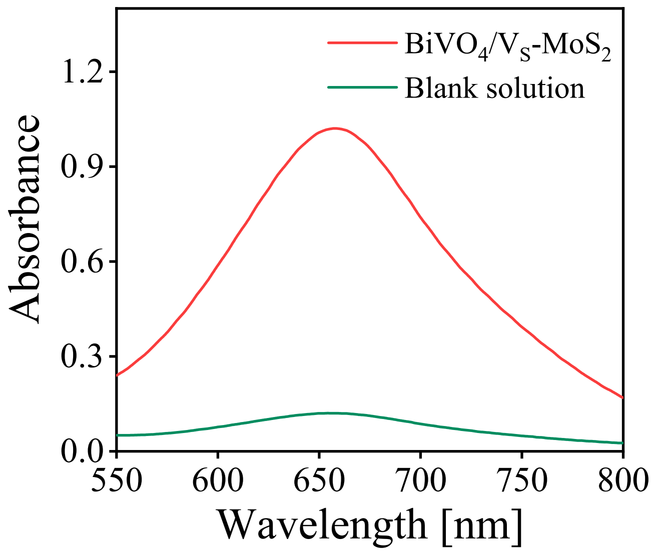

Fig. S12. The UV−Vis absorption spectra of blank solution and products solution with $BiVO_4/V_S$-MoS_2 as photocatalyst measured by indophenol blue method.

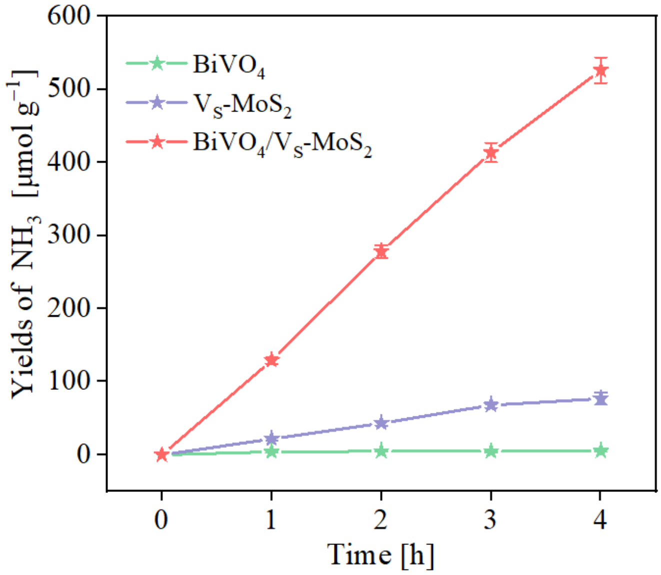

Fig. S13. The time-dependent photocatalytic NH_3 production of $BiVO_4$, V_S-MoS_2, and $BiVO_4/V_S$-MoS_2 composites.

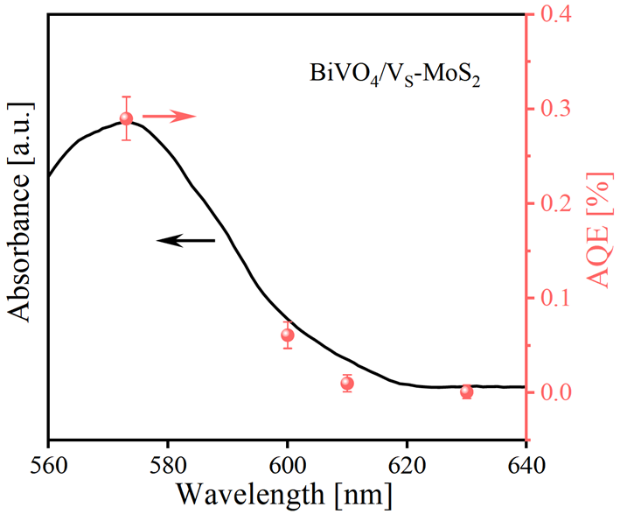

Fig. S14. The apparent quantum efficiency (AQE) of $BiVO_4/V_S$-MoS_2 for photocatalytic NH_3 production.

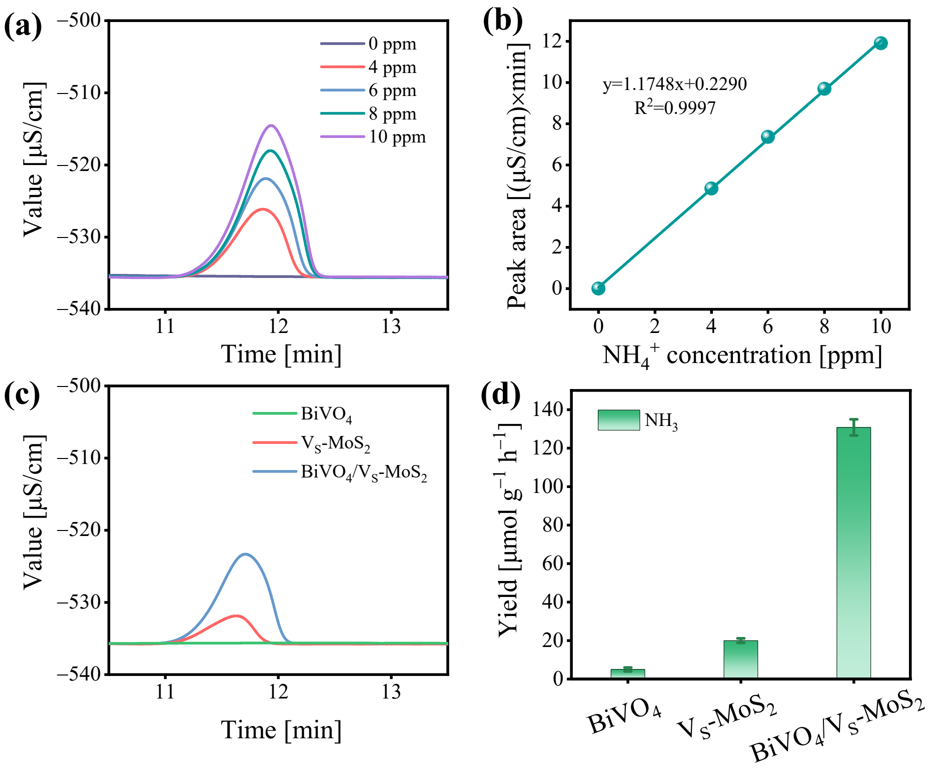

Fig. S15. (a) Ion chromatography of NH_4^+ solution with different concentrations, and (b) the corresponding calibration curve used for calculating the concentration of NH_4^+. (c) Ion chromatography of product solution after photocatalytic N_2 reduction with $BiVO_4$, V_S-MoS_2, and $BiVO_4$/V_S-MoS_2 as catalysts, respectively. (d) The yields of NH_3 production for $BiVO_4$, V_S-MoS_2, and $BiVO_4$/V_S-MoS_2.

As shown in Fig. S15c and 15d, pure $BiVO_4$ has nearly no NH_3 synthesis activity, and V_S-MoS_2 has a weak NH_3 activity (19.1 ± 1.3 μmol g^{-1} h^{-1}). $BiVO_4$/V_S-MoS_2 shows a significantly improved photocatalytic NH_3 synthesis performance of 130.7±3.9 μmol g^{-1} h^{-1}, nearly 7 times that of pure V_S-MoS_2. These results are in agreement with those obtained by the indophenol blue method.

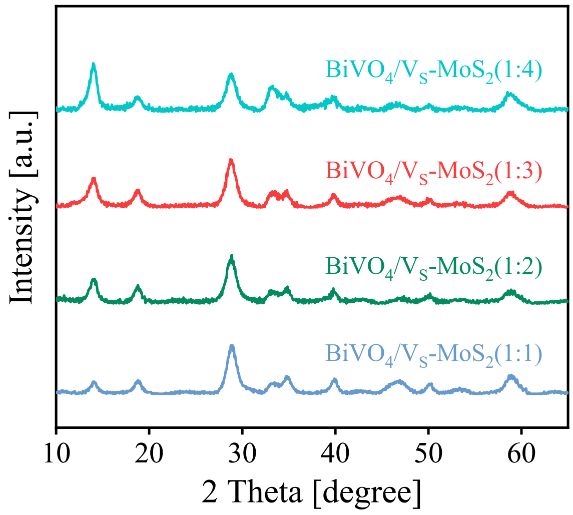

Fig. S16. The XRD patterns of $BiVO_4/V_S$-MoS_2 composites with different proportions.

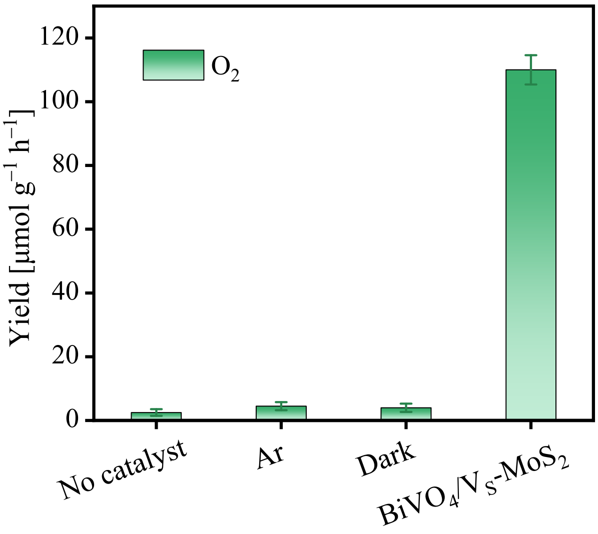

Fig. S17. The O_2 generation rates during photocatalytic reaction using $BiVO_4/V_S$-MoS_2 as photocatalyst under different conditions.

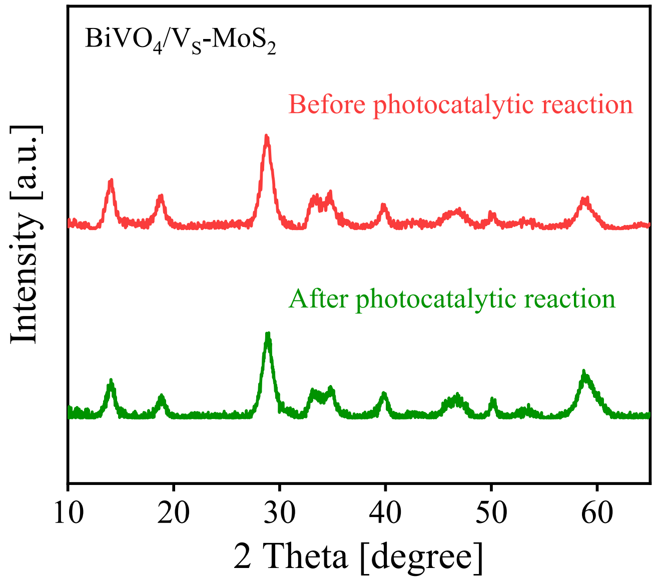

Fig. S18. The XRD patterns of $BiVO_4/V_S$-MoS_2 before and after photocatalytic reaction.

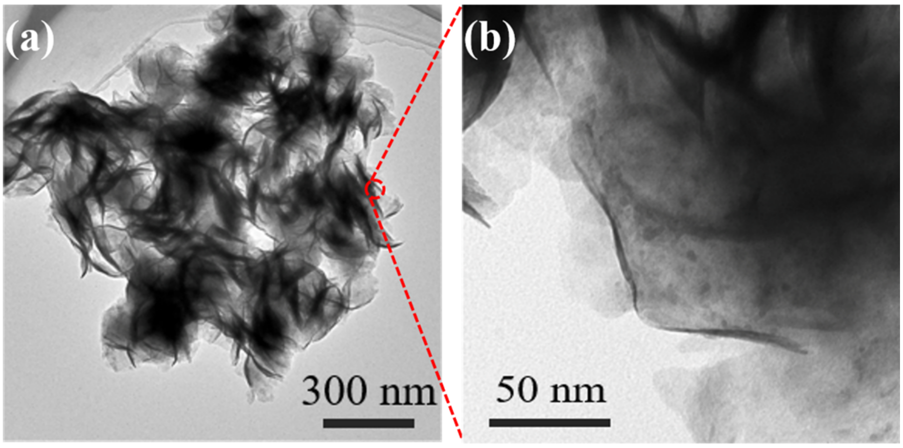

Fig. S19. The TEM images of $BiVO_4/V_S$-MoS_2 after photocatalytic N_2 fixation reaction.

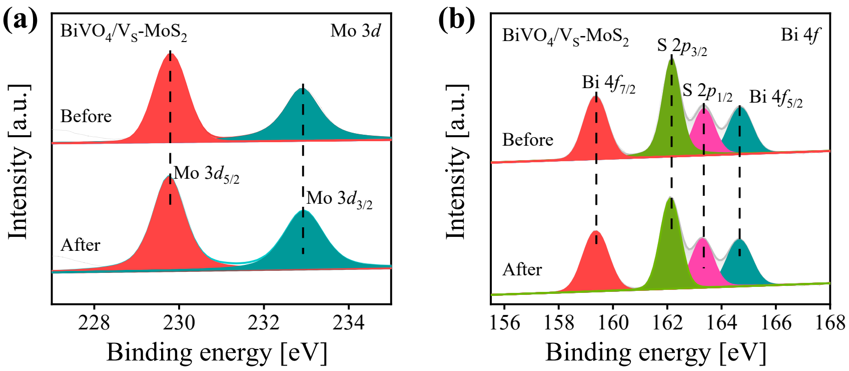

Fig. S20. High-resolution XPS spectra of $BiVO_4/V_S$-MoS_2 before and after photocatalytic N_2 fixation reaction. (a) Mo 3*d* and (b) Bi 4*f*.

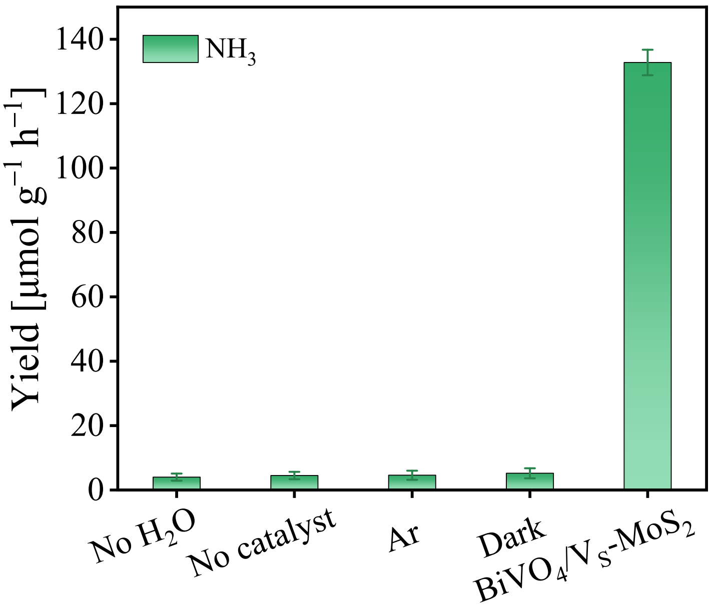

Fig. S21. Control experimental results under different conditions with $BiVO_4/V_S$-MoS_2 as photocatalytic catalyst.

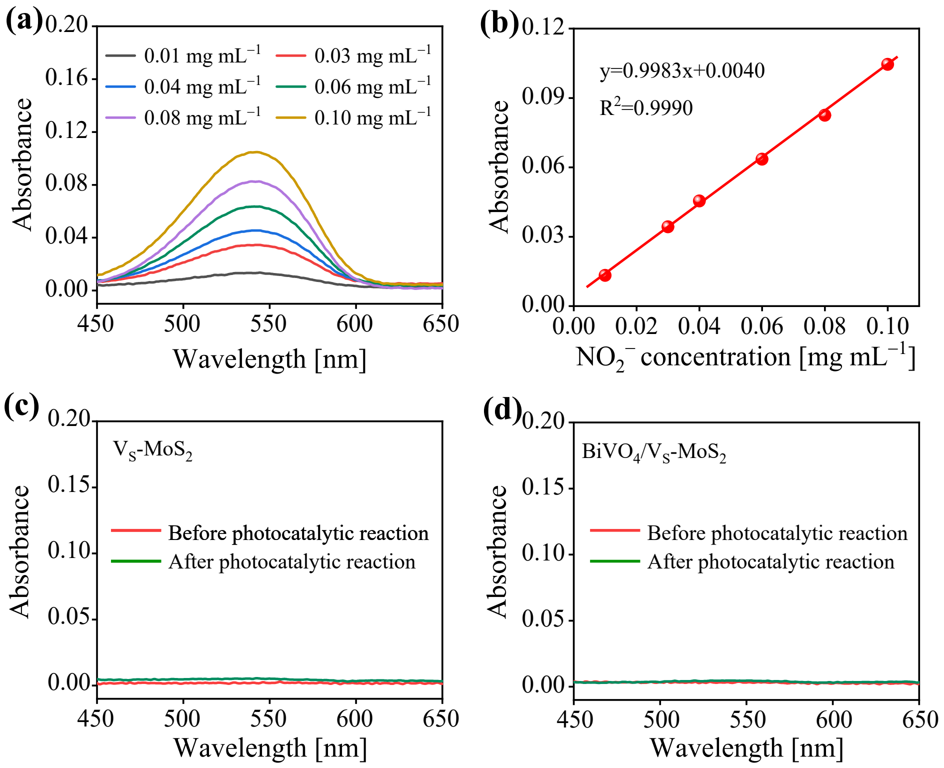

Fig. S22. (a) UV−Vis absorption spectra of NO_2^- solutions with different concentrations measured by colorimetric method, and (b) the corresponding calibration curve used for calculating the concentration of NO_2^-. (c, d) UV−Vis absorption spectra of product solutions measured by colorimetric method before and after photocatalytic N_2 reduction with V_S-MoS_2 and $BiVO_4$/V_S-MoS_2 as catalysts, respectively.

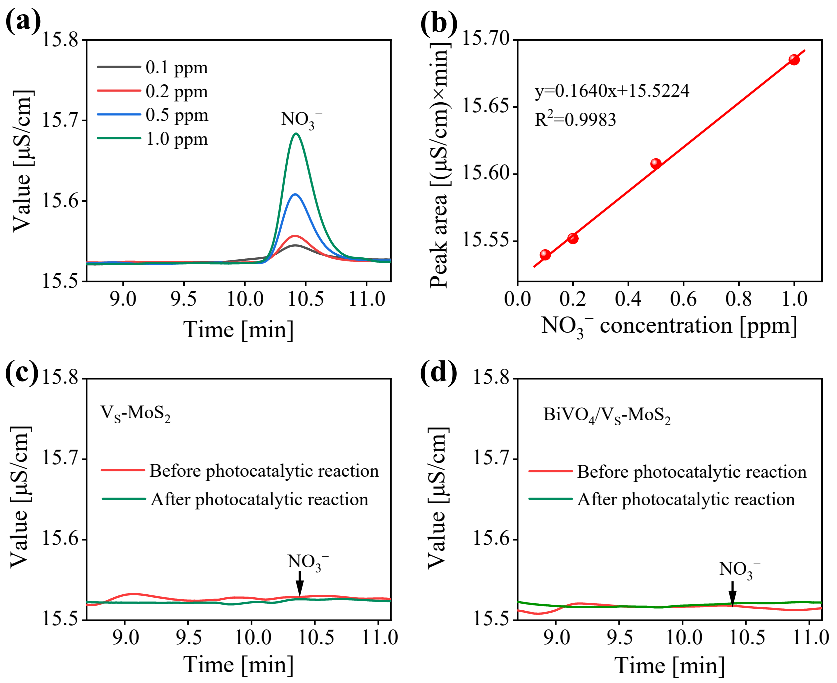

Fig. S23. (a) Ion chromatography of NO_3^- solution with different concentrations, and (b) the corresponding calibration curve used for calculating the concentration of NO_3^-. (c, d) Ion chromatography of product solution before and after photocatalytic N_2 reduction with V_S-MoS_2 and $BiVO_4/V_S$-MoS_2 as catalysts, respectively.

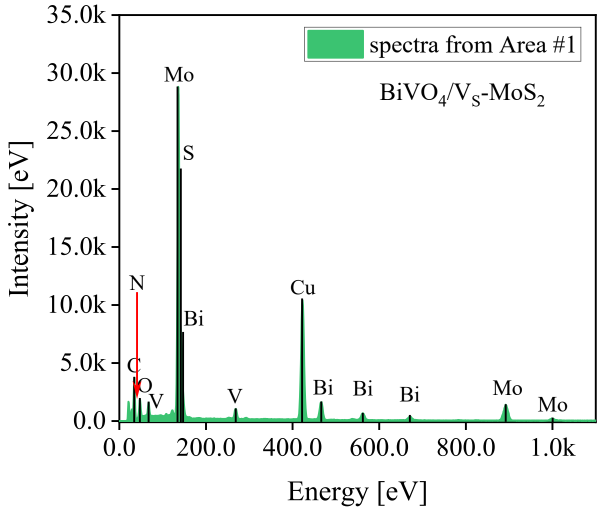

Fig. S24. EDS peaks of $BiVO_4/V_S$-MoS_2.

Table S2. The atomic percentages of $BiVO_4/V_S$-MoS_2 obtained by analyzing EDS elemental mapping.

Elements	Atomic Fraction (%)	Atomic Error (%)
N	0	NA
O	3.4	0.3
S	36.6	2.3
V	3.0	0.2
Mo	44.7	3.1
Bi	12.3	0.5

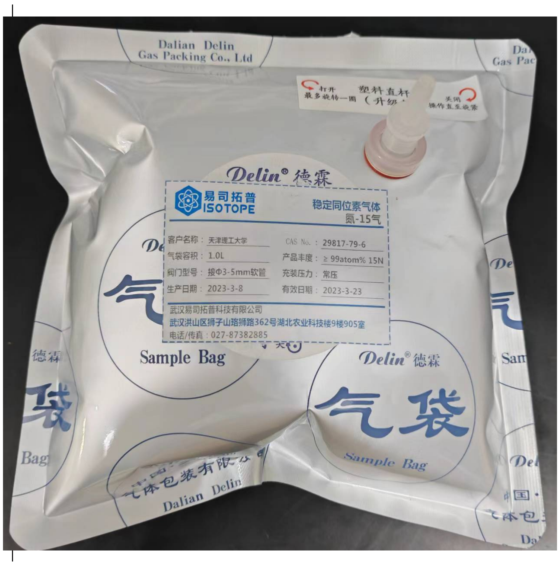

Fig. 25. The $^{15}N_2$ bag for isotope labeling tests.

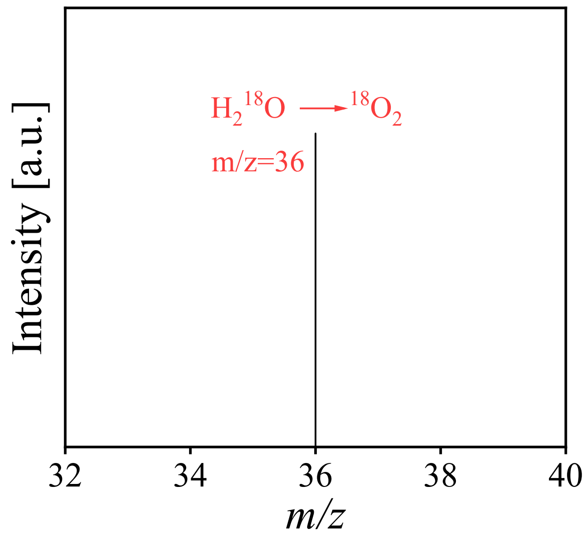

Fig. S26. MS analysis of photocatalytic $H_2{}^{18}O$ oxidation to $^{18}O_2$ (m/z = 36) using $BiVO_4/V_S$-MoS_2 as the photocatalyst.

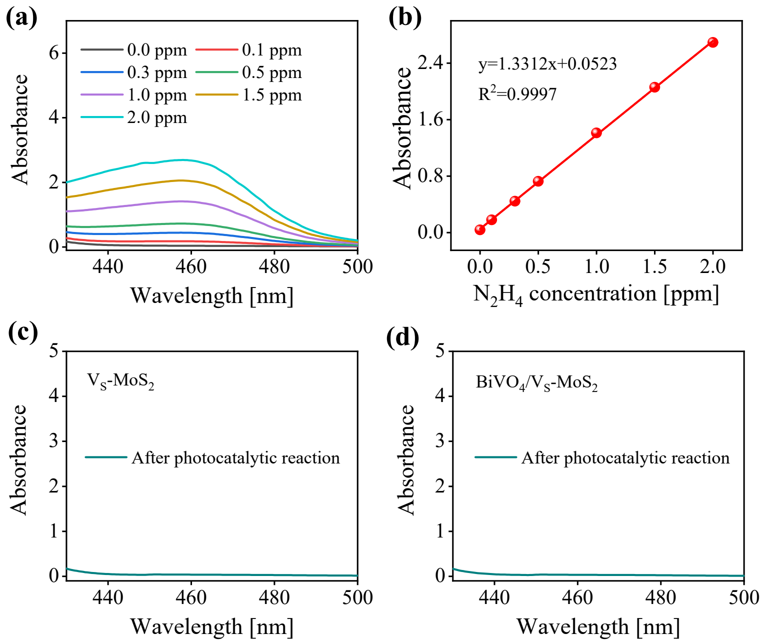

Fig. S27. (a) UV−Vis absorption spectra of N_2H_4 solutions with different concentrations measured by the Watt−Chrisp method (the details were described in the Methods section), and (b) the corresponding calibration curve. UV−Vis absorption spectra of product solutions measured by the Watt−Chrisp method with (c) V_S-MoS_2 and (d) $BiVO_4$/V_S-MoS_2 as photocatalysts.
